# Supplementary material for: Predicting visual function by interpreting a neuronal wiring diagram
Source: Nature. 2024 Oct 2;634(8032):113–23. doi: 10.1038/s41586-024-07953-5 (PMC11446822; doi:10.1038/s41586-024-07953-5)
Supplement: Supplementary file 6 — CRF and ERF predictions for individual TmY4 and TmY9 cells. Analogous to Supplementary Data 3, but for TmY target types. Shown are the top four monosynaptic pathways, the strongest pathway passing through each of the top ten intermediary types (ranking from Extended Data Fig. 7), and the trisynaptic pathway Tm1–TmY–Dm3–TmY (see the section entitled Prediction of spatial normalization). [file 41586_2024_7953_MOESM6_ESM.zip › DataS4/TmY9q/720575940629179291.pdf]

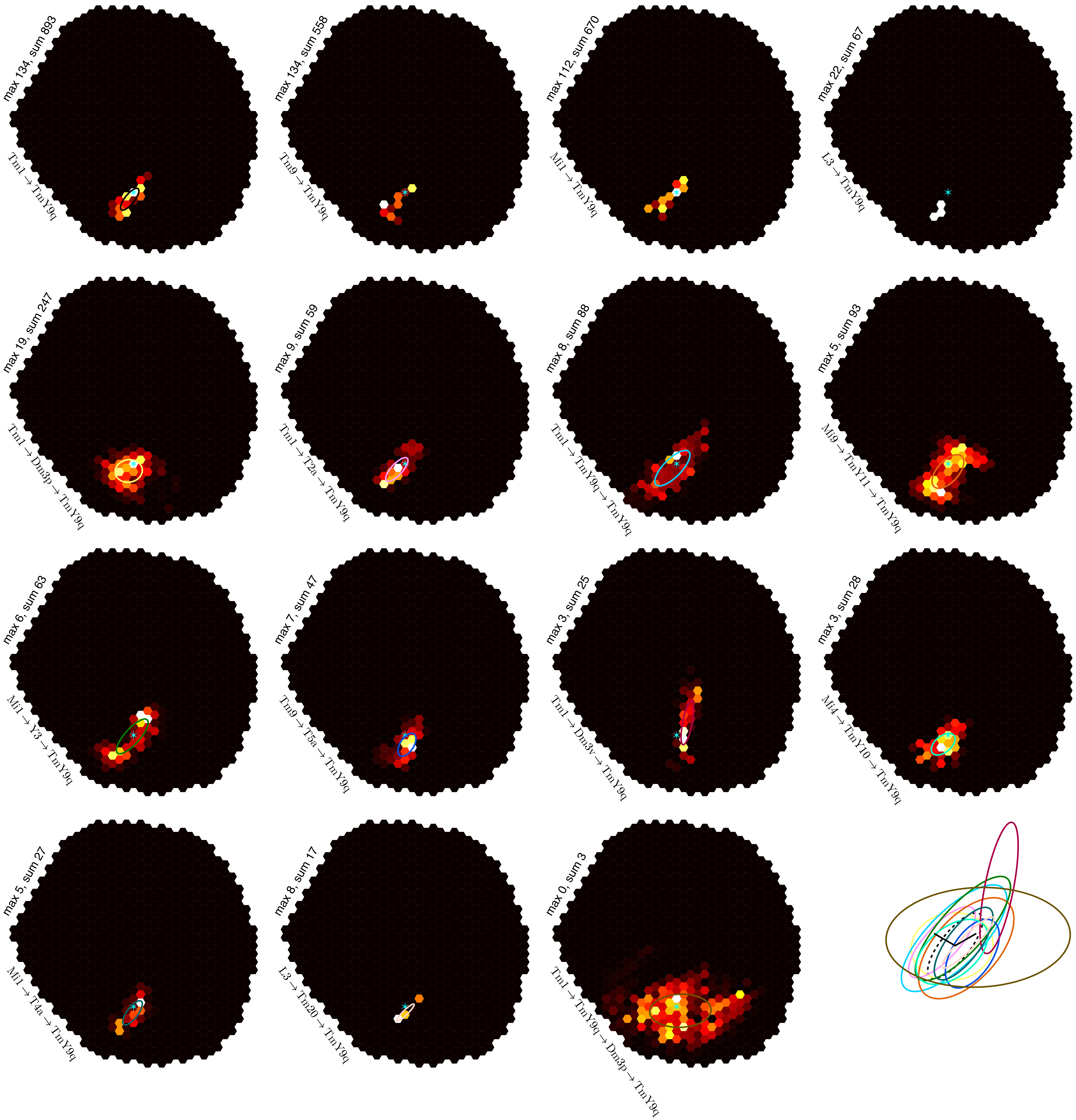

max 134, sum 893
Tm1 → TmY9q
max 134, sum 558
Tm9 → TmY9q
max 112, sum 670
Mi1 → TmY9q
max 22, sum 67
L3 → TmY9q
max 19, sum 247
Tm1 → Dm3p → TmY9q
max 9, sum 59
Tm1 → T2a → TmY9q
max 8, sum 88
Tm1 → TmY9q → TmY9q
max 5, sum 93
Mi9 → TmY11 → TmY9q
max 6, sum 63
Mi1 → Y3 → TmY9q
max 7, sum 47
Tm9 → T5a → TmY9q
max 3, sum 25
Tm1 → Dm3v → TmY9q
max 3, sum 28
Mi4 → TmY10 → TmY9q
max 5, sum 27
Mi1 → T4a → TmY9q
max 8, sum 17
L3 → Tm20 → TmY9q
max 0, sum 3
Tm1 → TmY9q → Dm3p → TmY9q
